# Neural Responses to Hypoxic Injury in a Vascularized Cerebral Organoid Model

**DOI:** 10.1007/s12264-025-01396-2

**Published:** 2025-04-22

**Authors:** Yang Li, Xin-Yao Sun, Peng-Ming Zeng, Zhen-Ge Luo

**Affiliations:** 1 School of Life Science and Technology & State Key Laboratory of Advanced Medical Materials and Devices, ShanghaiTech University, Shanghai, 201210 China; 2 Institute of Neuroscience, Center for Excellence in Brain Science and Intelligence Technology, Chinese Academy of Sciences, Shanghai, 200031 China

**Keywords:** Hypoxia, Hypoxic-injury encephalopathy, Cerebral organoid, Vascularized cerebral organoid, BMP signaling

## Abstract

Hypoxic injury (HI) in the prenatal period often causes neonatal neurological disabilities. Due to the difficulty in obtaining clinical samples, the molecular and cellular mechanisms remain unclear. Here we use vascularized cerebral organoids to investigate the hypoxic injury phenotype and explore the intercellular interactions between vascular and neural tissues under hypoxic conditions. Our results indicate that fused vascularized cerebral organoids exhibit broader hypoxic responses and larger decreases in panels of neural development-related genes when exposed to low oxygen levels compared to single cerebral organoids. Interestingly, vessels also exhibit neural protective effects on T-box brain protein 2^+^ intermediate progenitors (IPs), which are markedly lost in HI cerebral organoids. Furthermore, we identify the role of bone morphogenic protein signaling in protecting IPs. Thus, this study has established an *in vitro* organoid system that can be used to study the contribution of vessels to brain injury under hypoxic conditions and provides a strategy for the identification of intervention targets.

## Introduction

Hypoxic injury (HI) is the main cause of neonatal encephalopathy, and its incidence rate ranges from two to four per thousand [1–3]. Hypoxic-ischemic encephalopathy (HIE) is a condition that can develop at various stages of childbirth. It may begin during pregnancy due to issues like placental insufficiency or maternal health problems, such as cord prolapse, prolonged labor, intrauterine asphyxia, or respiratory distress, leading to brain injury due to oxygen deprivation [1, 4]. HIE can cause neurological disorders, including cerebral palsy and intellectual disabilities [5, 6]. To simulate neonatal HIE, animal models such as rodents, pigs, and sheep have been established [1, 2, 7, 8]. While animal models provide valuable insights into certain aspects of brain function, they fall short of mimicking the full complexity of the human brain. Therefore, alternative approaches are needed to study the responses of the human brain to hypoxic injury more comprehensively and accurately [2, 6, 9].

Stem cell technology and 3D cell culture systems have become increasingly mature, making it possible to simulate human brain development *in vitro* [10–12]. Based on these advances, cerebral organoids (Cors) have been developed to explore the mechanisms of developmental [13, 14], psychiatric [15], or neurodegenerative diseases [16, 17]. Several studies have used Cors to simulate HIE using different approaches [18–21]. One strategy has simulated HIE by exposure of Cors to hypoxia (1% O_2_ for 48 h); this later induced the specific impairment of T-box brain protein 2-positive (TBR2^+^) intermediate progenitors (IPs) [20]. Another approach has utilized ten-day-old Cors in 1% O_2_ and 5% CO_2_ for 72 h or 8% O_2_ and 5% CO_2_ for 25 days, showing that cortical markers such as Foxg1, Tbr1, and Ctip2 exhibit impaired expression during hypoxia compared to normal oxygen conditions (21% O_2_ and 5% CO_2_) [18, 19]. Another study suggests that short-term hypoxic treatment, in which cells are exposed to 3% O_2_ for 24 h followed by reoxygenation, can lead to a reduction in outer radial glia progenitors and other neural cells [19]. Although these studies have demonstrated the feasibility of using Cors as a model to simulate some HIE-related features of human neonates [22], they did not include vasculature or microglia, which are believed to play important roles in brain development and injury [23–28].

Recently, we have constructed fused vascularized cerebral organoids (FVCors) by fusing mesoderm-derived vessel organoids (Vors) with ectoderm-derived Cors [29, 30]. The model forms multiple cell types, such as endothelial cells and pericytes, constituting vascular network-like structures and a blood-brain barrier (BBB)-like structure. The FVCors exhibit an increased number of neural progenitors and contain active microglia. A recent study using the same approach has recapitulated the cavernoma structure and BBB disruption seen in patients [31].

In this study, we have utilized FVCors to explore the role of vessels in HI to neural parts. After 40 days of differentiation *in vitro*, the FVCors were exposed to a low oxygen concentration (0.6% O_2_, 5% CO_2_), following previous strategies [18, 20]. We found that neural cells in FVCors displayed a more intense hypoxic response than that in Cors, and vessels protected TBR2^+^ IPs from HI likely by releasing BMP2. Thus, this study provides an *in vitro* model mimicking HIE and demonstrates complicated intercellular interactions in neural responses to HI. The model established in this study demonstrates the potential to identify novel neural protection factors.

## Materials and Methods

### Cell Lines

Cells from the H9 human embryonic stem cell line (H9-hES) were purchased from iMedCell, and their identity was verified through STR profiling conducted by Applied Cell. Testing showed that the cells were negative for *Mycoplasma*. The H9-hES-EGFP line was created by integrating the CAG-EGFP DNA fragment into the ROSAβgeO26 genomic locus using the CRISPR/Cas9 gene-editing technique [30].

### hESC Culture

Both H9-hES and H9-hES-EGFP cell lines were maintained and cultured following the protocols described earlier [29, 32, 33]. The cells were grown on dishes coated with hESC-Matrigel (Corning) in StemFlex medium (Thermo) containing 4 ng/mL basic Fibroblast Growth Factor (Stemcell). The cells were passaged every 4 days using the ReLeSR passaging reagent (Stemcell).

### Generation of Cerebral Organoids and Treatments

The organoid culture method followed the procedure described in our recent report to generate cerebral organoids (Cors) and fused vascularized cerebral organoids (FVCors) [30]. Briefly, H9 ESCs with or without GFP (green fluorescent protein) labeling were used to generate brain-specific vessel organoids and cerebral organoids, respectively, following the steps of embryoid body (EB) induction, mesodermal and endothelial induction to generate vascular progenitors (VPs), or ectodermal and neural induction (for cerebral organoids). On day 12, neuroepithelial EBs (NE-EBs) were embedded in Matrigel for subsequent neural differentiation and maturation to generate Cors. Also on day 12, two VP-EBs were aligned with one NE-EB side-by-side embedded in Matrigel and cultured using the same medium for Cor induction with the addition of VEGF (20 ng/mL) to promote vascularization. At 40 days of differentiation, Cors and FVCors maintained in 21% O_2_ and 5% CO_2_ were transferred to a C-chamber hypoxia sub-chamber and incubated for 48 h. The oxygen level was controlled using an oxygen controller and a mixed CO_2_/N_2_ compressed gas source. After 48 h, Cors and FVCors were immediately collected for subsequent analysis. To determine the effects of BMP2, day 40 Cors were treated with 10 ng/mL BMP2 [Stemcell, 78004] for 48 h under 21% O_2_ and 5% CO_2,_ followed by hypoxia (0.6% O_2_) or normoxia (21% O_2_) for 48 h.

### Immunofluorescence

The organoids of interest were fixed in 4% paraformaldehyde (PFA) in PBS overnight at 4°C. The next day, the fixed organoids were dehydrated in 30% sucrose at room temperature for 4 h. Following dehydration, the organoids were embedded in the O.C.T. compound and cryosectioned at 30 μm. The sections were pre-treated with PBS-T and blocked for 1 h at room temperature in a humidified chamber in a solution containing 5% BSA and 0.1% Triton X-100 in PBS. Then, the sections were incubated with primary antibodies diluted in 5% BSA and 0.1% Triton X-100 in PBS at 4 °C for >48 h, followed by incubation with secondary antibodies diluted in 5% BSA and 0.1% Triton X-100 in PBS at 4 °C overnight in a humidifier chamber. The sections were subsequently embedded in PermaFluor, covered with a coverslip, sealed with clear nail polish, and stored at 4 °C until imaging. All images were captured using confocal imaging systems.

### Cell sorting and Flow Cytometry

Organoids were first treated with a trypsin solution to enzymatically break down the extracellular matrix to separate the cells. The reaction was carried out at 37 °C for 30 min until the cells were fully dissociated. After dissociation, the reaction was terminated by adding 2 mL 1% BSA in PBS to stop the enzymatic activity, and the resulting single-cell suspension was then centrifuged to remove excess trypsin before resuspending the cells in a fresh medium for further processing. To guarantee effective cell sorting using flow cytometry, the cell concentration was adjusted to ~1×10^6^ cells/mL.

Immediately before sorting, a 40-μm cell strainer was used to filter the cell suspension. To reduce cell adhesion during collection, the collection tubes were pretreated with 1 mL of 1% BSA in PBS. Throughout the sorting process, all samples and reagents were maintained on ice to preserve sample integrity. FlowJo software was used for subsequent data analysis.

### Quantitative PCR

Total RNA was extracted from the cells sorted by flow cytometry from 8–9 FVCors or Cors using TRIzol (Thermo/Life/Invitrogen), followed by reverse transcription to generate cDNA with the FastKing RT Kit (with gDNase; Tiangen). Quantitative PCR was applied using the AB Quanut Studio 7 Real-Time PCR System (Life Technologies) with 2×SYBR Green Mix (Bimake). Relative mRNA expression was assessed using the delta cycle threshold method, with human β-actin serving as the internal control for data normalization. The primer sequences used were as follows: *EOMES*: 5′- ACTGGTTCCCACTGGATGAG-3′ (forward), 5′-CCACGCCATCCTCTGTAACT-3′ (reverse); *PAX6*: 5′-TGGGCAGGTATTACGAGACTG-3′ (forward), 5′-ACTCCCGCTTATACTGGGCTA-3′ (reverse); *BCL11B*: 5′-CACCCCCGACGAAGATGACCAC-3′ (forward), 5′-AAA CTGGAACGGTGAAGG-3′ (reverse); *TBR1*: 5′-GTCACCGCCTACCAGAACAC-3′ (forward), 5′-ACAGCCGGTGTAGATCGTG-3′ (reverse); *BMP2*: 5′-GGAACGGACATTCGGTCCTT-3′ (forward), 5′-CACCATGGTCGACCTTTAGGA -3′ (reverse); *β-actin*: 5′-GTCTTTGCGGATGTCCAC-3′ (forward), 5′-AAACTG GAACGGTGAAGG-3′ (reverse).

### Bulk RNA sequencing

Total RNA was extracted using TRIzol according to the manufacturer’s instructions. For RNA sequencing, libraries were constructed using 500 μg total RNA. Raw sequencing reads were first evaluated for quality control using FastQC (version 0.10.1). The raw reads in FASTQ format were then subjected to adapter trimming and low-quality sequences were removed using Cutadapt (version 1.9.1). The preprocessed reads were mapped to the human genome (GRCh38) using Hisat2 (v2.0.1) with default parameters. Differentially expressed genes (DEGs) were identified using the R statistical package DESeq2, with a *P*_adjust_ value of 0.05 and a fold-change cutoff of ± 2. Functional enrichment of DEGs was carried out using Gene Ontology (GO) analysis using the clusterProfiler package in R (version 3.4.3). The volcano plots, heatmaps, and GO analysis plots were generated using R, incorporating the ggplot2 package for graphical representation.

### Statistical Analysis

Cell counts and fluorescence intensity were statistically analyzed using ImageJ 1.54f, followed by differential analysis using GraphPad Prism 10. qPCR results were pre-processed in Excel and then analyzed for differences using GraphPad Prism 10. The normality tests were first applied to each group of data; pairwise comparisons were then made using an unpaired *t*-test for normally distributed data, otherwise, the Mann-Whitney test was used. Data are presented as the mean ± SEM. *P < 0.05, **P < 0.01, ***P < 0.001.

## Results

### Establishing the HIE Model With Human Fused Vascularized Cerebral Organoids

**Fig. 1:**
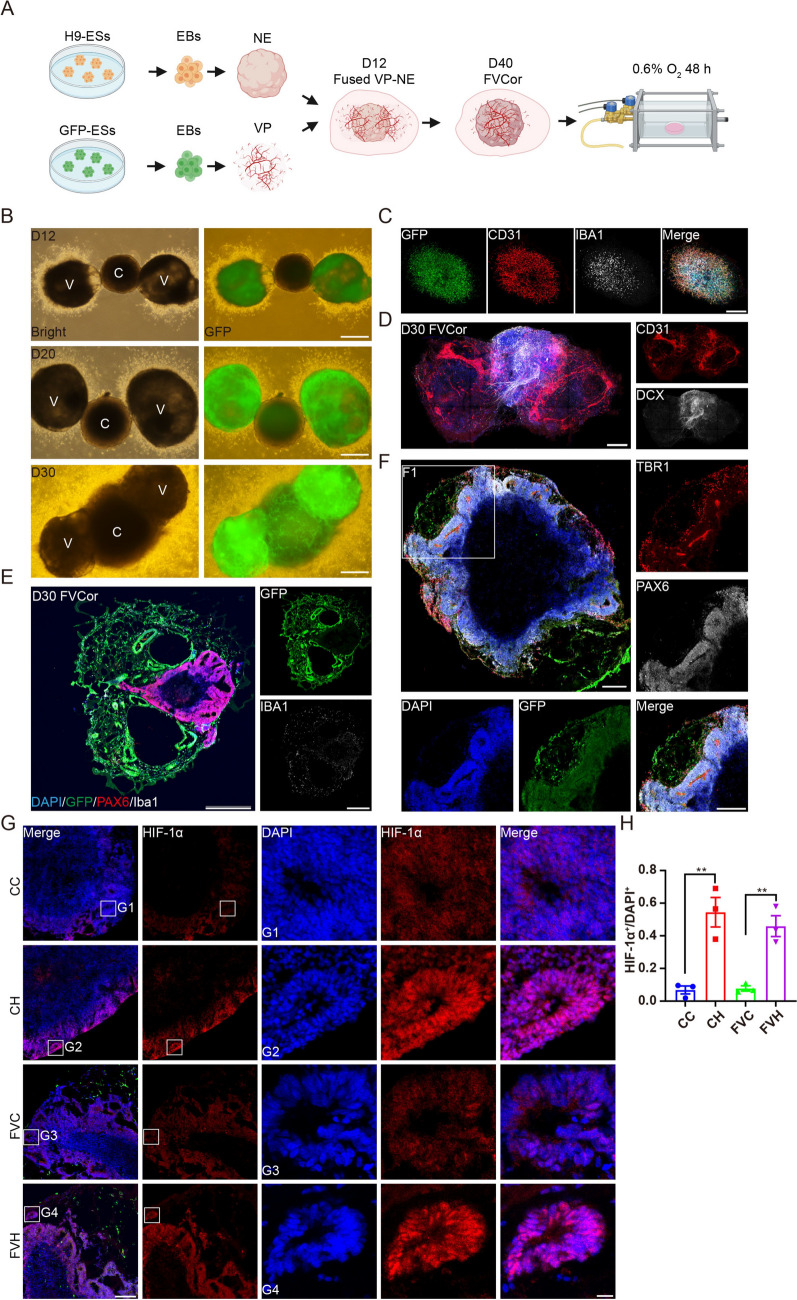
Hypoxia model in human fused vascularized cerebral organoids (FVCors). **A** Schematic of the major steps in generating FVCors from H9-ESCs and GFP-labeled ESCs. EBs, embryonic bodies; NE, neuroepithelium; VP, vascular progenitor. At day 40 (D40) of *in vitro* differentiation, FVCors are exposed to 0.6% O_2_ for 48 h in a gas-controlled culture chamber. Control FVCors are maintained at 21% O_2_ throughout. **B** FVCors at indicated developmental stages. Scale bars, 500 μm. V, Vessel organoid; C, Cerebral organoid. **C** Whole-mount staining of D20 GFP-labeled Vor, CD31, and IBA1 for labeling vessels and microglia. Scale bars, 500 μm. **D** Immunostaining of CD31 and doublecortin for labeling vessels and neurons, respectively, in D30 FVCors. Scale bars, 500 μm. **E** Immunostaining of GFP, PAX6, and IBA1 for labeling vessels, neural progenitors, and microglia, respectively, in D30 FVCors. Scale bars, 500 μm. **F** Immunostaining of GFP, TBR1, and PAX6 for labeling vessels, neurons, and neural progenitors in D40 FVCors. Scale bars, 500 μm. **F1**, enlarged area. Scale bar, 100 μm. **G** Representative staining of HIF-1α (red) in Cors and FVCors exposed for 48 h to 0.6% O_2_ *versus* 21% O_2_. Nuclei labeled by DAPI staining. Scale bar, 500 μm. G1, G2, G3, G4, enlarged areas. Scale bar, 50 μm. **H** Quantification of the density of HIF-1α^+^ cells. Data are presented as the mean ± SEM (*n =* 3 organoids). **P < 0.01, unpaired *t* test. CC, Cors Control; CH, Cors exposed to hypoxia; FVC, FVCors Control; FVH, FVCors exposed to hypoxia.

Because vessels are derived from mesoderm while neural tissue is derived from ectoderm, it is unrealistic to simultaneously induce both vessels and neural tissue from stem cells [34]. To create a reproducible experimental model of prenatal hypoxia during early human brain development, we applied the vascularized cerebral organoid protocol as we recently described [29, 30]. Using this protocol, we generated Cors and Vors separately and then fused them together. Firstly, Human ESCs were aggregated to generate EBs, which were then subjected to neuroectodermal induction and neural differentiation [10]. Meanwhile, GFP-labeled human ESCs were induced into EBs, followed by mesodermal induction and endothelial differentiation (Fig. 1A). In the separately cultured Vors on day 20 (D20), vascular structures composed of CD31^+^ endothelial cells and IBA1^+^ microglia were observed (Fig. 1C). Then, one Cor and two GFP-labeled Vors were placed in Matrigel droplets on D12 and then cultured under rotating conditions on an orbital shaker after 4 days (Fig. 1A). The whole-mount images of D20 and D30 fused vascularized cerebral organoids (FVCors) revealed the successful fusion of GFP-labeled Vors and Cors (Fig. 1B). The whole-mount staining images of D30 FVCors revealed that young doublecortin-labeled neurons were enwrapped by branched endothelial cells labeled by CD31 (Fig. 1D). Moreover, IBA1^+^ microglial cells derived from GFP^+^ ESCs persisted in FVCors on D30 and were in close contact with paired box 6 (PAX6)^+^ neural progenitors localized in neuroepithelial rosettes (Fig. 1E). By D40, neuroepithelium fate was successfully induced in the FVCors as reflected by the expression of PAX6 and cortical layer marker T-box brain transcription factor 1 (TBR1) (Fig. 1F). At ~D40 of differentiation, FVCors were placed in a gas control chamber and incubated under low oxygen tension (0.6% O_2_) for 48 h (Fig. 1A). Immunofluorescent staining in FVCors slices revealed that hypoxia-inducible factor-1 alpha (HIF-1α), a critical oxygen-labile protein in the hypoxia pathway [6, 35, 36], was induced after exposure to low oxygen (Fig. 1G, H), indicating successful induction of the hypoxia response.

### More Robust Responses in Fused Vascularized Cerebral Organoids After Hypoxia

**Fig. 2:**
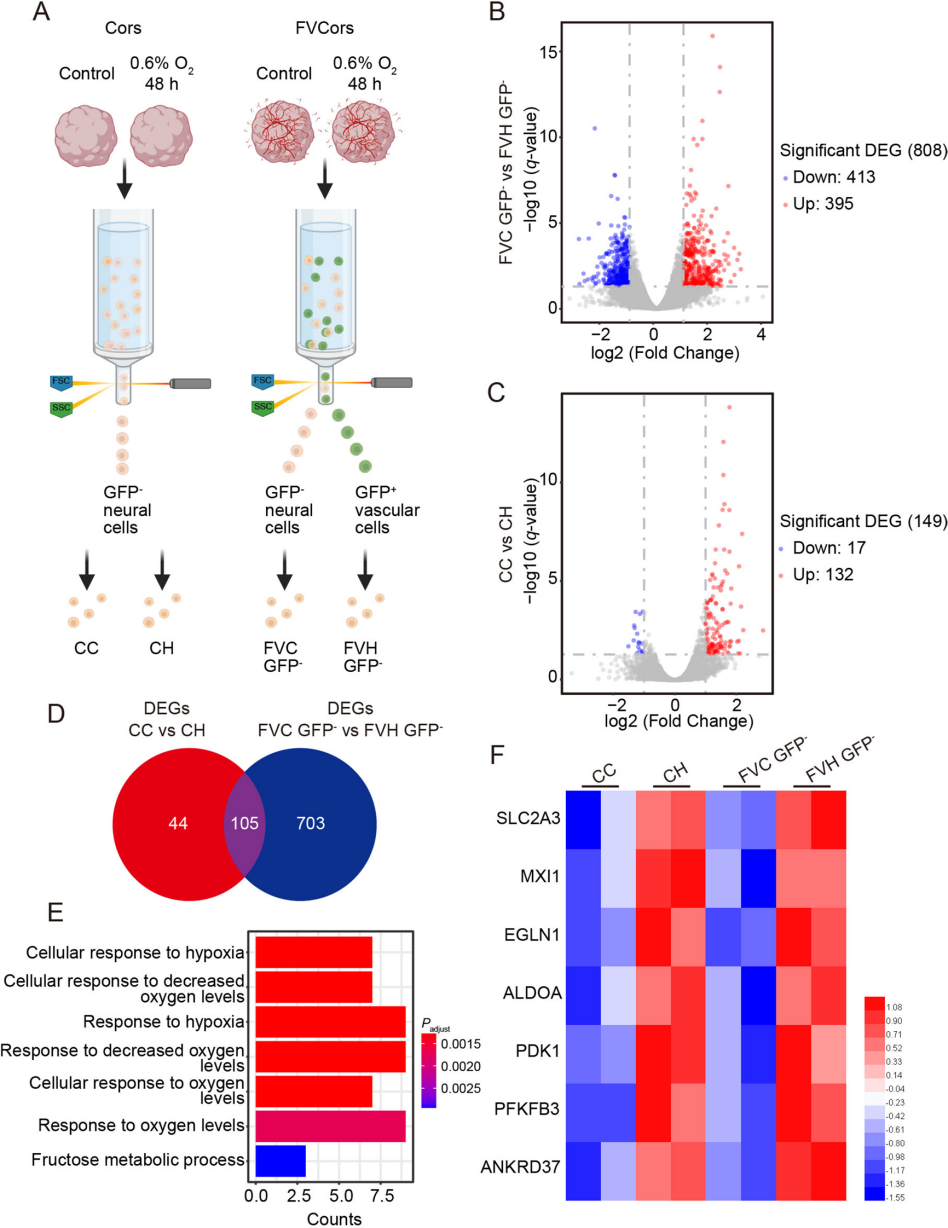
Hypoxic responses induced in FVCors and Cors. **A** Schematic of isolation of GFP^-^-neural cells from control and hypoxic FVCors for transcriptomic analysis. **B** Volcano plots showing differentially- expressed genes (DEGs) of GFP^-^ neural cells from FVCors after exposure for 48 h to 0.6% O_2_ *versus* 21% O_2_. Each dot represents a single gene; red, significantly upregulated; blue, significantly downregulated; gray, no significant difference (fold change >2, *P*_adjust_ <0.05). **C** Volcano plots showing DEGs of Cors after exposure to 0.6% O_2_ for 48 h *versus* 21% O_2_. Each dot represents a single gene; red, significantly upregulated; blue, significantly downregulated; gray, and no significant difference (fold change >2, *P*_adjust_ <0.05). **D** Venn diagram showing the overlap of DEGs in Cors (149) after exposure to 0.6% O_2_ for 48 h and FVCors (808) after exposure to 0.6% O_2_ for 48 h. **E** Gene Ontology analysis of the 105 overlapped genes in D (*P*_adjust_ <0.05). **F** Heatmap of the hypoxia pathway-related DEGs across the four groups. Red/blue, higher/lower relative expression levels. CC, Cors Control; CH, Cors exposed to hypoxia; FVC, FVCors Control; FVH, FVCors exposed to hypoxia.

To identify hypoxia-related signaling pathways of neural cells in FVCors after exposure to low oxygen, we isolated GFP-negative neural cells from normoxic (21% O_2_) control FVCors and hypoxic FVCors using fluorescence-activated cell sorting (Fig. 2A). We identified DEGs in neural cells in FVCors exposed to two conditions (0.6% O_2_ and 21% O_2_) using bulk RNA sequencing (RNA-seq). In total, we identified 808 DEGs (Fig. 2B; *P*_adjust_ <0.05, |fold change| >2). To determine the influence of vessels on neural components, we applied a similar analysis to non-assembled Cors at the same stages after exposure to normoxic and hypoxic conditions. Surprisingly, 149 DEGs were identified between control and hypoxic Cors (Fig. 2C; *P*_adjust_ <0.05, |fold change| >2). Notably, 105 DEGs (hypoxia *vs* normoxia) were shared and 747 DEGs were distinct between Cors and FVCors (Fig. 2D). GO analysis revealed that most shared DEGs, including *SLC2A3* and *PDK1* [37–39], were associated with the hypoxia response pathway, suggesting that exposure to low oxygen results in a defined transcriptional profile (Fig. 2E, F). The marked increase in DEGs between control and hypoxic FVCors suggested that FVCors have a more intense response to hypoxia than Cors (Fig. 2D).

**Fig. 3:**
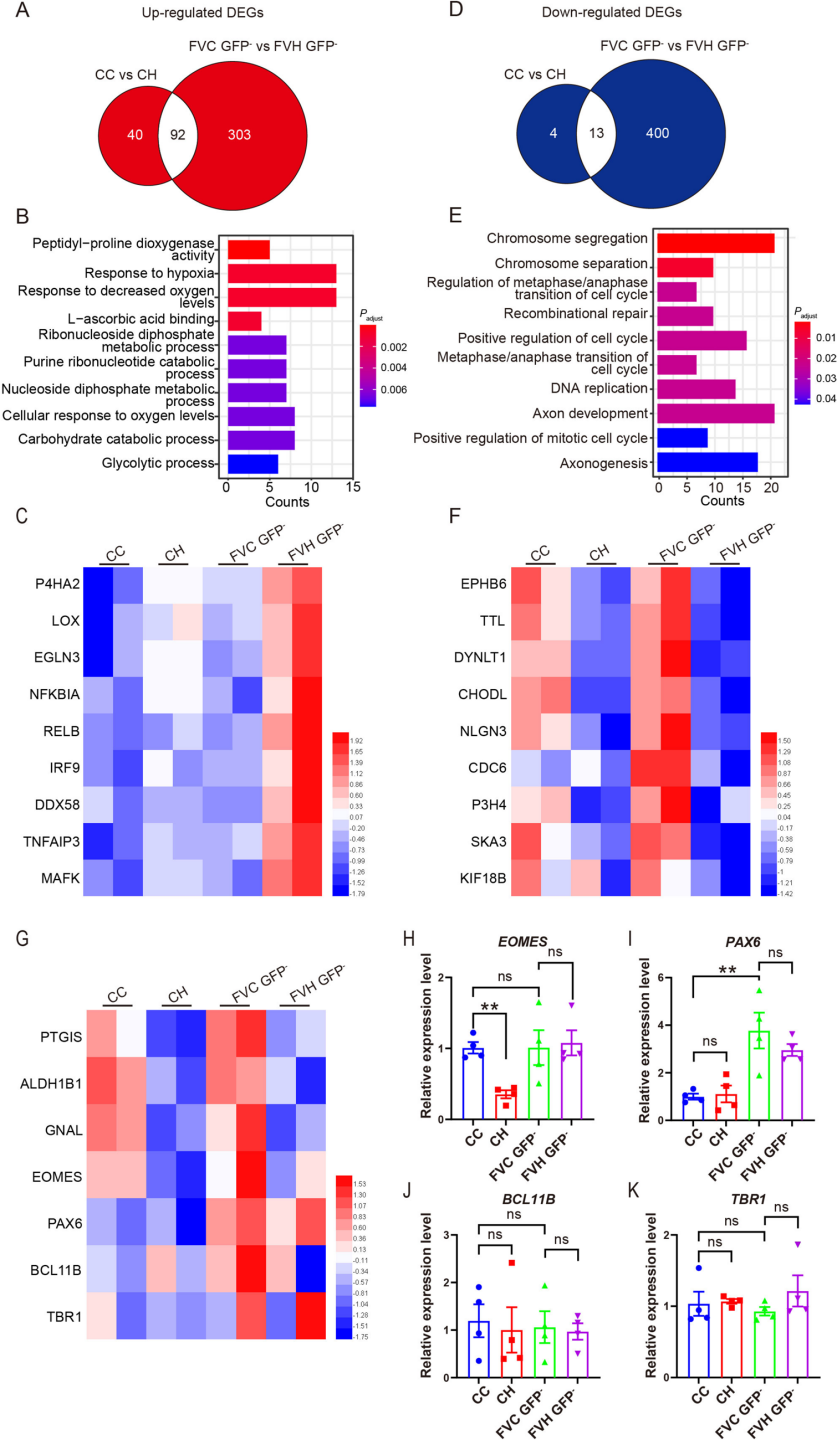
More robust responses in FVCors exposed to hypoxia than in Cors. **A** Venn diagram of the up-regulated DEGs in Cors after hypoxia and FVCors after hypoxia. **B** GO analysis of the 343 DEGs in **A** (*P*_adjust_ <0.05). **C** Heatmap of protein hydroxylation and inflammation-related DEGs. Red/blue, higher/lower relative expression levels. **D** Venn diagram of the down-regulated DEGs in Cors after hypoxia and FVCors after hypoxia. **E** GO analysis of the 404 different DEGs in **D** (*P*_adjust_ <0.05). **F** Heatmap of DEGs of FVC GFP^-^ *vs* FVH GFP^-^ associated with chromosome segregation and axon development. Red/blue, higher/lower relative expression levels. **G** Heatmap of down-regulated DEGs in CC *vs* CH and several neuron markers. Red/blue, higher/lower relative expression levels. **H–K** qPCR analysis of several neural markers. Relative expression is normalized to β-actin. Data are presented as the mean ± SEM of four independent experiments, each involving 8–10 organoids per group. **P < 0.01, unpaired *t* test. CC, Cors Control; CH, Cors exposed to hypoxia; FVC, FVCors Control; FVH, FVCors exposed to hypoxia.

**Fig. 4:**
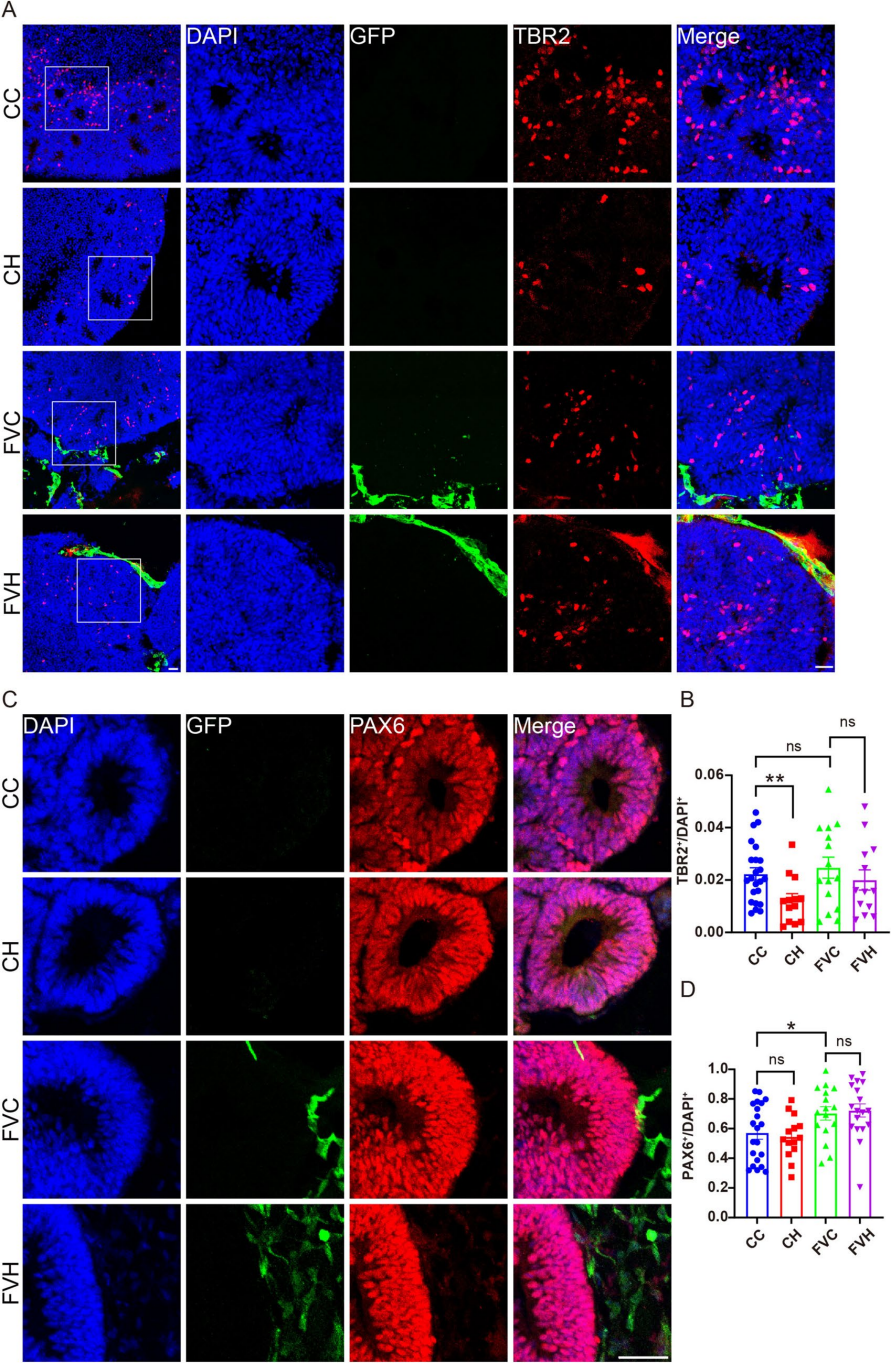
TBR2^+^ IP cell numbers do not decrease in FVCors after exposure to hypoxia. **A, C** IF staining of GFP, TBR2, and PAX6 to label vessels, IPs, and neural progenitors, respectively, in D40 FVCors. Scale bars, 50 μm. **B, D** The density of TBR2^+^ (**B**) and PAX6^+^ (**D**). Data are presented as the mean ± SEM (TBR2: *n =*15–23 rosettes from 3–4 organoids; PAX6: *n = *14–22 rosettes from 3–4 organoids). *P < 0.05, **P < 0.01, unpaired* t* test. CC, Cors Control; CH, Cors exposed to hypoxia; FVC, FVCors Control; FVH, FVCors exposed to hypoxia.

To further compare the response of neural cells in FVCors to hypoxia with that of non-assembled Cors, we compared the up-regulated DEGs of neural cells in FVCors to Cors exposed to hypoxia and found that the 303 distinct up-regulated DEGs were associated with hypoxia and metabolic process-related pathways (Fig. 3A, B). After the analysis of DEGs, we noted that inflammation-related genes, such as *RELB*, *IRF9*, and *TNFAIP3*, which have been reported to play crucial roles in HIE [40, 41], were significantly up-regulated in hypoxic FVCors, while they were not changed in hypoxic non-assembled Cors (Fig. 3C). Then we compared the down-regulated DEGs of neural cells in FVCors *vs* Cors exposed to hypoxia and found that the 404 distinct down-regulated DEGs were enriched in pathways associated with chromosome segregation and cell-cycle progression, which have been shown to be the primary manifestations following hypoxia injury [42–44] (Fig. 3D–F). Moreover, FVCors exposed to hypoxia showed significantly reduced gene expression associated with axon development and axonogenesis, consistent with axon injury phenotypes in neonatal brain injury models [6, 45] (Fig. 3E, F). In line with the previous report that hypoxic treatment reduces the number of IPs [20], the expression of *EOMES*, the gene encoding T-box brain protein 2 (TBR2), was significantly reduced in Cors after exposure to hypoxia (Fig. 3G, H). However, this decrease was less pronounced in neural cells of hypoxic FVCors (Fig. 3G, H). Consistent with this result, the density of TBR2^+^ IPs was reduced in Cors but not FVCors after 48 h under hypoxia (0.6% O_2_) (Fig. 4A, B). By contrast, hypoxia had no effect on the expression of *BCL11B* or *TBR1*, in either Cors or FVCors (Fig. 3J, K). In agreement with our previous results [29], FVCors exhibited greater production of PAX6^+^ neural progenitors (NPs) than Cors as reflected by increased *PAX6* expression (Fig. 3I) and the density of PAX6^+^ cells, but this increase was not dampened by hypoxia (Fig. 4C, D). Thus, the FVCors exhibit complicated responses to hypoxia, including detrimental and beneficial effects on neural cells.

### Vessels Alleviate the Decrease of TBR2^+^ Intermediate Progenitors (IPs) by BMP2

**Fig. 5:**
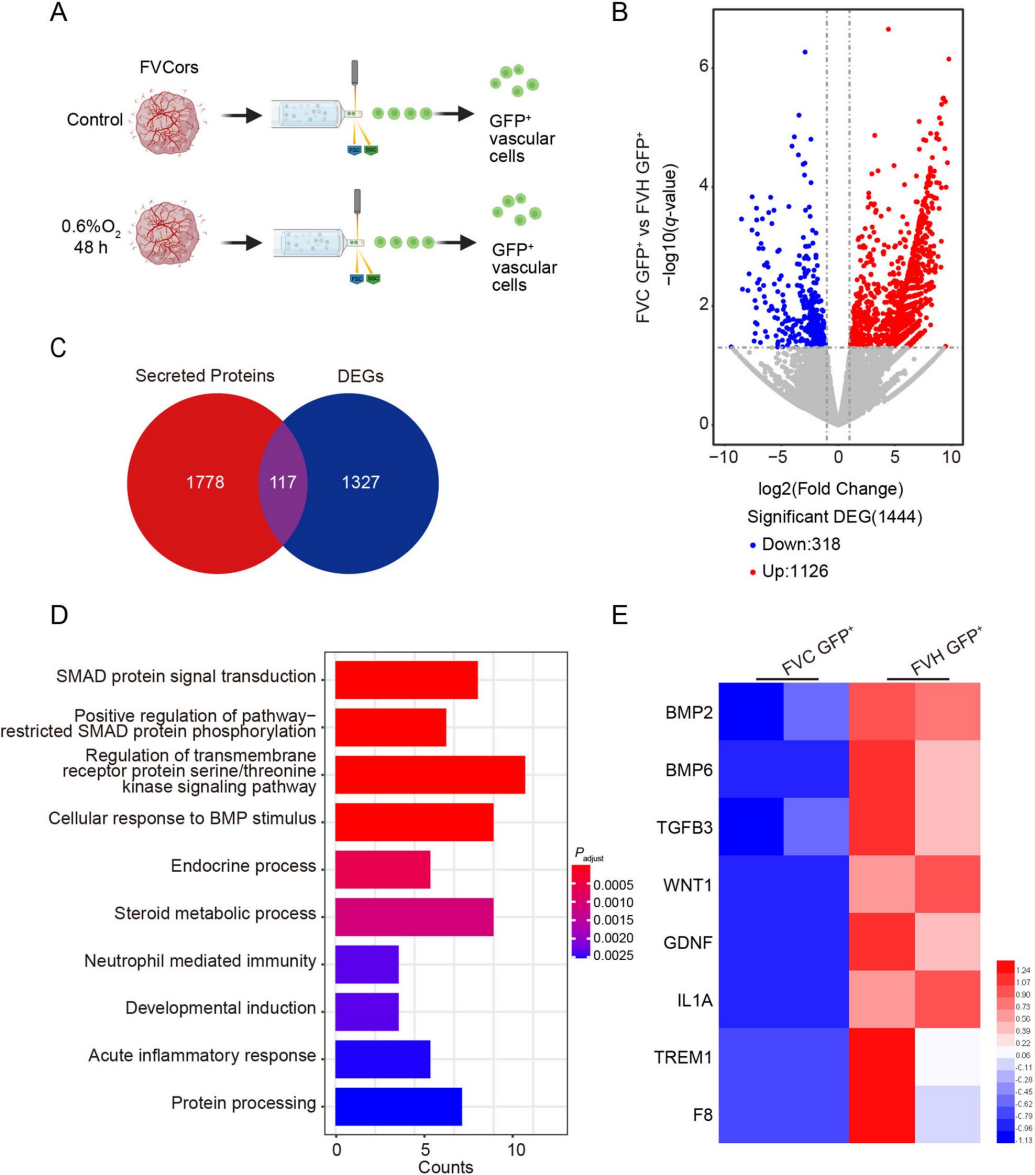
Transcriptomic analysis of GFP^+^ vascular cells isolated from control and hypoxic FVCors. **A** Schematic of the isolation of GFP^+^ vascular cells from control and hypoxic FVCors for transcriptomic analysis. **B** Volcano plots depicting DEG analysis of vascular cells from control and hypoxic FVCors. Each dot represents a single gene; red, significantly upregulated; blue, significantly downregulated; gray, no significant difference (fold change >2, *P*_adjust_ <0.05). **C** Venn diagram showing the overlap of DEGs in vascular cells from control and hypoxic FVCors and the secreted protein dataset (data from v24.0.proteinatlas.org). **D** GO analysis of the 117 overlapped DEGs in **C** (*P* <0.1 and *P*_adjust_ <0.05). **E** Heatmap of up-regulated secreted DEGs in FVC GFP^+^ *vs* FVH GFP^+^. Red/blue, higher/lower relative expression levels. FVC GFP^+^, GFP^+^ vessels of FVCors Control; FVH GFP^+^, GFP^+^ vessels of FVCors exposed to hypoxia.

**Fig. 6:**
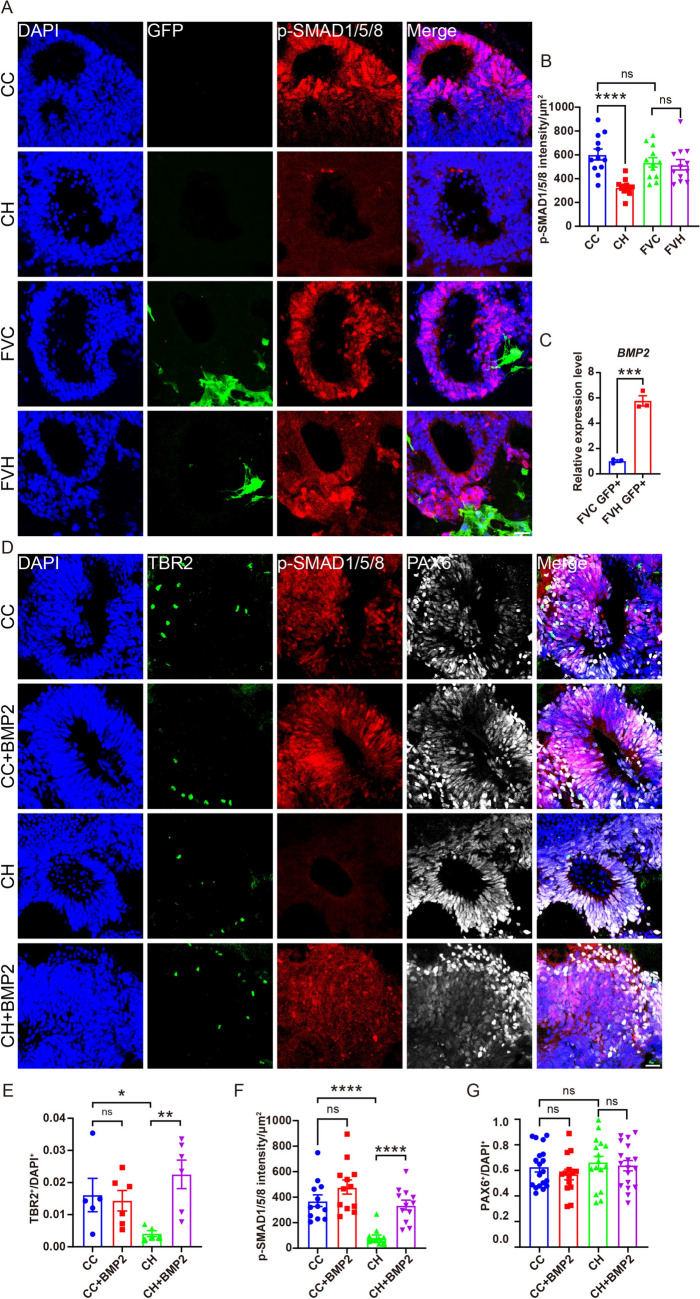
BMP2 ameliorates the hypoxia-induced TBR2 decrease in Cors. **A** Confocal images of p-SMAD1/5/8 signals in indicated organoids. Scale bar, 50 μm. **B** qPCR analysis of the expression of BMP2 in vascular cells from control and hypoxic FVCors. Relative expression is normalized to β-actin. Data are presented as the mean ± SEM of three independent experiments, each involving 8–10 organoids per group. ***P < 0.001, unpaired *t* test. **C** Quantification of the intensity of p-SMAD1/5/8. Data are presented as the mean ± SEM (*n = *12 rosettes from 3 organoids). ****P <0.0001, unpaired *t* test. **D** Confocal images demonstrating TBR2, p-SMAD1/5/8, or PAX6 signals in hypoxic Cors with BMP2 treatment. Scale bar, 50 μm. **E, G** Quantification of the TBR2^+^ (**E**) and PAX6^+^ (**G**) density. Data are presented as the mean ± SEM (TBR2: *n = *5–6 rosettes from 3–4 organoids; PAX6: *n = *19–22 rosettes from 3–4 organoids). *P < 0.05, **P < 0.01, unpaired *t* test. **F** Quantification of the intensity of p-SMAD1/5/8. Data are presented as the mean ± SEM (*n =*12 rosettes from 3 organoids). ****P <0.0001, unpaired *t* test. CC, Cors Control; CH, Cors exposed to hypoxia; FVC, FVCors Control; FVH, FVCors exposed to hypoxia; FVC GFP^+^, GFP^+^ vessels of FVCors Control; FVH GFP^+^, GFP^+^ vessels of FVCors exposed to hypoxia.

To gain further insights into how the vascular cells of FVCors work to ameliorate TBR2^+^ IP injury during hypoxia, we sorted GFP^+^ vascular cells in FVCors after exposure to hypoxia for RNA-seq and DEG analysis (Fig. 5A, B). Given that most vascular structures are wrapped around the peripheral regions of Cors, we speculated that vascular cells may exert their impact on neural cells through secreted factors. Thus, we focused on DEGs that are present in the secreted proteins dataset (data from v24.0.proteinatlas.org). The GO analysis showed 117 candidate genes, which presumably encode secreted proteins (Fig. 5C). Interestingly, from the enriched pathways, we found that the inflammatory response was activated in the vascular cells of hypoxic FVCors, which is consistent with the increase of inflammation-related genes in neural cells of hypoxic FVCors (Fig. 5D, E). This phenomenon demonstrated that FVCors exposed to hypoxia-activated the overall immune system through the activation of immune cells in vascular cells. In addition, many pathways were associated with bone morphogenetic protein (BMP) signaling and intracellular SMAD signal transduction, including *BMP2*, *BMP6*, *TGFB3*, and *WNT1* (Fig. 5D, E). Therefore, we hypothesized that the BMP-SMAD signaling pathway might be involved in the vascular protection of TBR2^+^ IPs under hypoxic conditions. We examined the phosphorylation levels of its downstream molecules SMAD1/5/8 to test this hypothesis. Immunostaining analysis of Cors sections revealed a decrease in the levels of phosphorylated SMAD1/5/8 after exposure to hypoxia, suggesting a decrease in BMP signaling (Fig. 6A, B). However, this tendency was markedly dampened in FVCors (Fig. 6A, B), indicating that the suppression of BMP signaling in FVCors under hypoxic conditions is relatively weak.

In line with this notion, we found that *BMP2* was indeed increased in the vascular cells of FVCors following hypoxic treatment (Fig. 6C). To determine whether the impairment in BMP/SMAD signaling contributes to the loss of IPs in Cors, we administered BMP2 during hypoxic treatment of Cors. We found that BMP2 markedly rescued the SMAD signaling and alleviated the decrease in TBR2^+^ IPs caused by hypoxia (Fig. 6D–F). Notably, PAX6^+^ cells were not affected, suggesting the specific effects of hypoxia and BMP2 on IPs (Fig. 6D, G).

Together, these results highlight the intricate relationship between BMP2 and TBR2 cell survival under hypoxic conditions, providing valuable insights into potential therapeutic strategies for neuroprotection in low-oxygen environments.

## Discussion

Previous studies using Cors derived from the ectoderm have been used to investigate the effects of hypoxia on neurogenesis [18–21]. However, the technique of inducing Cors *in vitro* with culture media and molecules limited the production of vascular tissues and microglia that originate from the mesoderm. In clinical and animal experiments, hypoxic injury can lead to the release of inflammatory cytokines by microglia, which induce apoptosis and cell death, thereby causing brain damage. Meanwhile, hypoxic environments can induce angiogenesis, alleviating tissue hypoxia. Moreover, some angiogenic proteins have been shown to protect against neural damage [4, 8, 26, 40]. Therefore, the presence of vascular structures and microglia is essential for studying the mechanisms of hypoxic brain injury. In this study, we subjected FVCors (including neural cells, vessels, and microglia) to 0.6% O₂ for 48 h, aiming to construct an HIE model that more closely resembles the *in vivo* environment. Using this model, we obtained hypoxic brain injury phenotypes that are more akin to HIE and enable the study of the interactions of vessels and microglia with neural cells, providing new targets for the treatment of hypoxic brain diseases.

Our results indicate that FVCors exhibit more complex gene alterations in response to hypoxia than the single Cors, including both beneficial and detrimental responses (Fig. 3), reflecting the intricate hypoxic response occurring *in vivo*. Previous studies have shown that inflammatory responses during HIE can expand the area of brain damage and worsen the severity of injury [40]. We found that FVCors exhibit inflammatory responses under hypoxic conditions, with a respective upregulation of inflammatory factors in vascular tissue and neural cells (Figs 3C, 5D), consistent with the literature [4]. Moreover, FVCors demonstrate downregulation of chromosomal segregation, cell-cycle regulation, and axonogenesis-related genes in response to hypoxic injury compared to non-assembled Cors (Fig. 3F), aligning more closely with the effects of hypoxia on DNA stability [42] and axonal guidance [45] *in vivo*. Overall, the vascularized brain organoids after hypoxic treatment exhibit a phenotype similar to *in vivo* hypoxic injury, which is likely due to the activation of microglia derived from vascular tissue, which may have triggered immune responses. This is consistent with *in vivo* studies that suggest inflammatory factors in hypoxic injury are one of the key contributors to brain damage [46]. The axonogenesis defects in hypoxic FVCors are more likely a combined result of both the stress and the inflammatory environment.

In addition to these detrimental effects, hypoxia can also induce vascular proliferation [47, 48], which alleviates neuronal damage [8]. Besides providing oxygen and nutrients, proteins secreted by blood vessels can influence neurogenesis and protect against neuronal injury [8]. We found that with the incorporation of vascular tissue, the reduction in IPs of the hypoxic non-assembled Cors was alleviated by BMP2 (Fig. 6), which is known to be part of a protein family that exerts various actions in neural pattering, fate determination, and neural protection, as well as detrimental effects on neurogenesis depending on cellular contexts [49, 50]. Indeed, the IP-reduction phenomenon is consistent with a previous study [20].

Despite these promising findings, several limitations must be acknowledged. Firstly, our model lacks the inclusion of blood cells and blood flow, which are critical components for ensuring the physiological and functional relevance of the vessels within the organoids. In addition, the maturity level of these organoids is still relatively low; they may only partially mimic the cell types and/or status of the brain *in vivo*. Further improvements are needed, including the inclusion of blood flow models, the development of more mature organoids, and comprehensive validation using animal models. Strategies to achieve long-term stability in organoid cultures will be crucial for extended studies. Nevertheless, the vascularized brain organoid model provides a unique window to obtain insights into brain development and pathology, and a feasible system to understand intercellular interactions. Given the heterogeneity across species, the organoids derived from hESCs have an advantage in revealing human-specific physiological and/or pathological features.

## Data Availability

The RNA-seq data of isolated neural cells and vascular cells have been deposited at NCBI with the accessible number PRJNA1185146. Other datasets generated and/or analyzed during the current study are available from the corresponding author upon reasonable request.
